# Wright’s Hierarchical *F*-Statistics

**DOI:** 10.1093/molbev/msae083

**Published:** 2024-05-02

**Authors:** Marcy K Uyenoyama

**Affiliations:** Department of Biology, Duke University, Box 90338, Durham, NC 27708-0338, USA

**Keywords:** identity coefficients, IBD, IBS, effective number, selfing

## Abstract

This perspective article offers a meditation on $F_{ST}$ and other quantities developed by Sewall Wright to describe the population structure, defined as any departure from reproduction through random union of gametes. Concepts related to the *F*-statistics draw from studies of the partitioning of variation, identity coefficients, and diversity measures. Relationships between the first two approaches have recently been clarified and unified. This essay addresses the third pillar of the discussion: Nei’s $G_{ST}$ and related measures. A hierarchy of probabilities of identity-by-state provides a description of the relationships among levels of a structured population with respect to genetic diversity. Explicit expressions for the identity-by-state probabilities are determined for models of structured populations undergoing regular inbreeding and recurrent mutation. Levels of genetic diversity within and between subpopulations reflect mutation as well as migration. Accordingly, indices of the population structure are inherently locus-specific, contrary to the intentions of Wright. Some implications of this locus-specificity are explored.

## Introduction

Among the fundamental descriptors of genetic variation in structured populations are Wright’s hierarchical *F*-statistics. Here, population structure corresponds to any departure from random union of gametes, including regular inbreeding and geographical subdivision of the gamete pool. Notably, $F_{ST}$ appears throughout the evolutionary literature (see accompanying virtual issue). As one might expect for such a widely used quantity, $F_{ST}$ serves a broad array of uses, including as a measure of distance between groups.

Sewall Wright (1921, 1923) introduced the *F*-statistics at the very inception of the field of population genetics and worked to clarify their meaning through subsequent decades (e.g. Wright 1943, 1951, 1965, 1977). This arena of evolutionary theory featured a collision of multiple conceptual approaches, primary among them the partitioning of variance, probabilities of identity by descent (IBD), and diversity. A few quotes may serve to convey a flavor of the discussion.

*F* was … proposed as an inbreeding coefficient giving “the departure from the amount of homozygosis under random mating toward complete homozygosis” … It has been used since as a measure of such departure relative to a specified foundation stock, not necessarily random bred. (Wright 1951, p. 325)

… $F_{ST}$ or Θ was introduced as the correlation between uniting gametes relative to those across all subdivisions … The concept of “relative to” is not an easy one, and it has made the study of population structure difficult. (Weir 2012, p. 638)

… gene diversity is defined by using the gene frequencies at the present generation, so that no assumption is required about the pedigrees of individuals, selection, and migration in the past. (Nei 1977, p. 225)

Weir and Goudet (2017) have provided a masterful unification of the variance partitioning and IBD approaches, clarifying ideas and approaches and summarizing the massive literature devoted to the theoretical aspects alone. Addressed here is the third pillar of thought on the definition and interpretation of $F_{ST}$: the approach through genetic diversity, especially indices associated with Nei’s $G_{ST}$ (Nei 1973, 1977).

Wright developed the *F*-statistics within the context of pedigrees extending few generations into the past relative to the age of segregating genetic variants. In this context, genes belonging to the same allelic class can be assumed to be identical by descent (IBD), meaning derived through an unbroken series of Watson–Crick replication events from a single gene held by an ancestor at the head of the pedigree. Here, identity by state (IBS) describes genes that belong to the same allelic class, with the definition of an allelic class highly context-dependent (see note on terminology at the end of this section).

Upon moving from his position at the United States Department of Agriculture as Senior Animal Husbandman to take a faculty position at the University of Chicago, Wright generalized pedigree-based approaches and concepts to encompass evolutionary biology (Crow 1994). Across evolutionary timescales, the observation of IBS between genes does not necessarily imply IBD, although as a practical matter, inferences about IBD have always derived from observations of IBS. Furthermore, depending on whether one’s notion of descent excludes mutation, the strong inference of IBD does not necessarily imply the observation of IBS.

This article begins with a review of an early study that provides some insight into the patterns that motivated Wright to develop the *F*-statistics and then rephrases Nei’s (1977) hierarchy of genetic diversity measures in terms of IBS probabilities. Explicit expressions for the IBS probabilities and $G_{ST}^{*}$, Nei’s analog of $F_{ST}$, are determined for the island model with regular inbreeding and a two-deme model with asymmetric migration and coalescence rates. Beyond the substitution of probabilities of IBD by probabilities of IBS, the shift in perspective compels a consideration of the origin of observed genetic variation as well as its pattern.

An explicit statement regarding terminology may facilitate communication among the various components of the large literature in this area. Here, *θ* denotes the scaled mutation rate fundamental to population genetics:


$$\theta =\underset{\underset{u\to 0}{N\to \infty }}{\text{lim}}4Nu,$$

while Θ denotes IBD probabilities or associations among genes (see Cockerham 1967, 1973). Furthermore, *gene* here refers to a set of nucleotides passed from parent to offspring, while *allele* is a shortened form of allelomorph, in the sense of Bateson and Saunders (1902). Alleles or allelic classes represent flavors of genes that might segregate in a population at a given location (locus) in the genome. For example, Mendel attributed the relative proportions of round and wrinkled peas observed in controlled crosses to segregation of the dominant *R* allele and recessive *r* allele. Allelic classes for major histocompatibility loci may reflect functional differences, with higher fitness associated with greater heterozygosity. For a single nucleotide site regarded as a locus, each kind of nucleotide segregating at the site in the population may be defined as an allelic class.

## Population Structure in the Island Model

Wright (1943, pp. 124–126) characterized hierarchical measures of differentiation in a population subdivided into a number of “intermediate” groups, each of which comprise a number of breeding groups undergoing random mating. Using the expected variance in allele frequency as a basis for comparison, he expressed the relative excess diversity among demes in the form


(1) $$\frac{F_{t}-F_{i}}{1-F_{i}}=\frac{\left(1-F_{i})-(1-F_{t}\right)}{1-F_{i}}$$

(Wright 1943, equation (43)), for $F_{i}$ and $F_{t}$ measures of genetic association at the levels of the intermediate groups and the total population, respectively.

A description of the context in which Wright developed the hierarchical *F*-statistics may serve to clarify their meaning (see Chapter 16 of Wright 1978). Wright’s analysis of the pedigrees of British Shorthorn cattle registered in the Coates herd book illustrates the conceptual foundation and provides an example of a hierarchy in gene diversity.

### Case Study

Wright (1923) studied the history of key lines of British Shorthorn cattle developed by brothers Charles and Robert Colling, who were among the earliest adopters of selective breeding techniques. Individual bovines bred by the Collings became widely admired celebrities, including Favourite, considered the greatest stud of its day, and its son Comet, the first bull to break the milestone of the 1,000 guinea selling price. Thomas Bates, another influential breeder, established a direct maternal line from a cow sired by Comet. Wright noted that the relatively low fertility of this highly prized Duchess line only increased market prices.

**Fig. 1.**
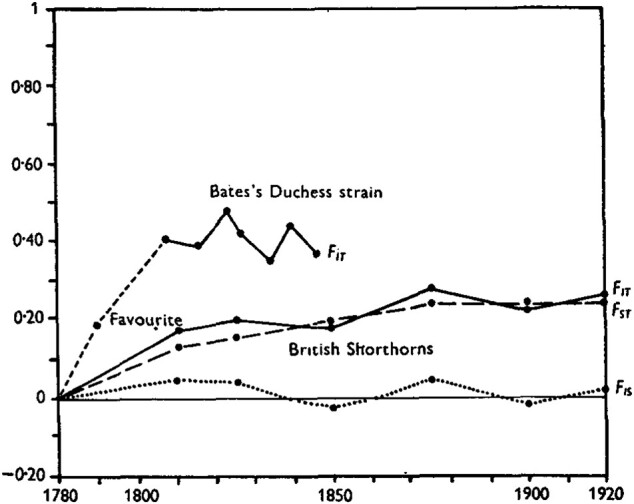
Hierarchical *F*-statistics for British Shorthorn pedigrees (Wright 1951). Reprinted with permission.

Figure 1 illustrates Wright’s application of the hierarchical *F*-statistics to the complex history of intense inbreeding in the British Shorthorns over the first 40 years of the Coates herd book (Wright 1923; McPhee and Wright 1925; Wright 1951). By convention in pedigree studies, members of the base population of 1780 are considered non-inbred and unrelated to one another, with $F_{IT}$ corresponding to the correlation between uniting gametes accumulated over the subsequent generations. For the celebrated stud Favourite, Wright (1923) computed a value of $F_{IT}$ of 0.192: it was nearly as inbred as the product of a parent-offspring mating ($1/2^{2}$). Thomas Bates’s Duchess line, descended from Favourite, shows even higher levels of inbreeding, with declines reflecting deliberate outcrossing.

The line labeled $F_{IS}$ appears to correspond to


(2) $$F_{IS}=\frac{\left(1-G_{w})-(1-F_{IT}\right)}{1-G_{w}}=\frac{F_{IT}-G_{w}}{1-G_{w}},$$

in which $F_{IT}$ now represents the average correlation between uniting gametes of registered Shorthorn cattle and $G_{w}$ the correlation between a pair of gametes randomly sampled from across the breed. Periods with positive values of $F_{IS}$ reflect the formation of offspring through the fusion of gametes more highly correlated than random gametes ($F_{IT}>G_{w}$), and negative values indicate gametes less correlated than random ($G_{w}>F_{IT}$).

Of particular interest is $F_{ST}$, represented by the dashed line in Fig. 1. Wright (1943, 1951, 1965, 1977) has described $F_{ST}$ as the correlation between uniting gametes for hypothetical offspring that would be produced by the institution of a single generation of random mating. Among those offspring, $F_{IS}^{\prime }=0$ and $F_{IT}^{\prime }=G_{w}$ (with $G_{w}^{\prime }=G_{w}$), which implies


(3) $$F_{ST}=G_{w}.$$

This expression together with (2) implies the iconic partitioning


(4) $$1-F_{IT}=(1-F_{IS})(1-F_{ST})$$

(e.g. Wright 1943, after his Equation (48)).

Note that this notion of $F_{ST}$ is distinct from Wright’s observation regarding the relationship between $F_{ST}$ and the Wahlund (1928) variance among allele frequencies. Wright (1969, p. 295) explains that in cases in which reproduction proceeds by random union of gametes in all demes and the demes are completely isolated from one another (absence of gene flow), $F_{IT}$ for the entire assemblage of non-communicating demes corresponds to the Wahlund variance. As $F_{IS}=0$ within demes, the partitioning (4) implies that $F_{ST}=F_{IT}$ corresponds to the Wahlund variance as well. Nei (1965) provides an explicit demonstration. This scenario does not apply to the British Shorthorns, for which the population comprises interlocking webs of reproduction rather than discrete, isolated demes.

### Hierarchical Structure

An enduring source of confusion surrounding the hierarchical *F*-statistics concerns which components are probabilities and which are correlations, whether they are scaled relative to an underlying demographic structure, and how that structure is defined. An explicit description of a hierarchy similar to that discussed by Nei (1977) may serve to clarify the discussion.

#### Hierarchy of IBS Probabilities

Let $h_{i}$ denote the probability of observing non-identity by state (non-IBS) between a pair of genes randomly sampled from level *i* of the hierarchy, without regard to the structure at any lower level. For level *i*, a measure of association analogous to the *G*-statistics of Nei (1977) corresponds to


(5) $$1-G_{i}^{*}=\frac{h_{i-1}}{h_{i}}.$$

At every level, the measure of association represents the probability of non-identity *relative to* the probability of non-identity on the next lower level. Rearrangement in the form of (1),


$$G_{i}^{*}=\frac{h_{i}-h_{i-1}}{h_{i}},$$

emphasizes the analogy between the $G_{i}^{*}$ and Wright’s (1951) panmictic indices (complements of the *F*-statistics).

Extension across an arbitrary number (*L*) of levels of organization produces


(6) $$\prod_{i=1}^{L}(1-G_{i}^{*})=\prod_{i=1}^{L}\frac{h_{i-1}}{h_{i}}=\frac{h_{0}}{h_{L}}.$$

Identifying the analog of $\left(1-F_{IT}\right)$ with this product implies


(7) $$1-G_{IT}^{*}=\frac{h_{0}}{h_{L}}.$$

These expressions mirror those given by Nei (1977, see his (22)), but with non-IBS probabilities replacing average indices of gene diversity ($\bar{H}$).

#### Wright’s Island Model

A fundamental starting point for the study of population structure, Wright’s island model postulates *d* demes, each with *N* diploid reproductives. Let *f* represent the probability of observing IBS between the pair of gametes that united to form a random individual. Similarly, $g_{w}$ denotes the probability of IBS between genes randomly sampled from distinct individuals residing in the same deme and $g_{b}$ the IBS probability between genes randomly sampled from distinct demes. While the notation evokes the Malécot coefficients (Malécot 1969; Jacquard 1974), *f*, $g_{w}$, and $g_{b}$ here refer to IBS in an evolving population and not to IBD in a pedigree.

The base level of the hierarchy refers to a pair of gametes that united to form a random zygote. Those genes represent distinct allelic classes with probability


$$h_{0}=1-f.$$

At the next level (demes comprising *N* reproductive zygotes), $h_{1}$ refers to a gene pair sampled from a given deme without regard to the structuring at the base level (genes organized into zygotes):


$$\begin{array}{rl} h_{1} & =[(1+f)/2]/N+(1-g_{w})(1-1/N)\\ & =1-g_{w}+O(1/N). \end{array}$$

At this level of the hierarchy (5),


(8) $$\begin{array}{cc} & 1-G_{1}^{*}=\frac{h_{0}}{h_{1}}=\frac{1\;-\;f}{1\;-\;g_{w}}+O(1/N),\\ & G_{1}^{*}=\frac{\;f\;-\;g_{w}}{1\;-\;g_{w}}+O(1/N). \end{array}$$

To large terms (>O(1/N)), $G_{1}^{*}$ represents the level of IBS within individuals relative to their local deme (Wright 1951). It is the analog of $F_{IS}$ (2).

Proceeding to the next level, the non-IBS probability between a pair of genes sampled from the metapopulation without regard to structuring at lower levels (demes or zygotes) corresponds to


$$\begin{array}{rl} h_{2} & =h_{1}/d+(1-g_{b})(1-1/d)\\ & =(1-g_{w})/d+(1-g_{b})(1-1/d)+O(1/N), \end{array}$$

where 1/d is the probability that the gene pair derive from a single deme. At this level of the hierarchy (5),


(9) $$\begin{array}{rl} & 1\;-\;G_{2}^{*}=\frac{h_{1}}{h_{2}}=\frac{1\;-\;g_{w}}{\left(1\;-\;g_{w})/d+(1\;-\;g_{b})(1\;-\;1/d\right)}+O(1/N)\;\\ & G_{2}^{*}=\frac{\left(1\;-\;1/d)(g_{w}\;-\;g_{b}\right)}{\left(1\;-\;g_{w})/d+(1\;-\;g_{b})(1\;-\;1/d\right)}+O(1/N). \end{array}$$

This last expression is the analog of Nei’s reformulation of $F_{ST}$, later called Nei’s $G_{ST}$ (equation (18) in Nei 1977).

Weir and Goudet (2017) explicitly acknowledge that their allele-based $F_{ST}$ ($\beta_{WT}$) departs from Wright’s formulation in its comparison of within-deme association to between-deme association (analogous to replacing $h_{2}$ by $\left(1-g_{b}\right)$). They indicate that their formulation is simpler in that relative deme sizes need not be specified. Under the island model, which assumes uniform deme size, $G_{2}^{*}$ is analogous to $\beta_{WT}$ in populations comprising many demes (d→∞).

In general, a hierarchy might well include structures beyond those considered to this point. An analysis of the British Shorthorns, for example, might address relationships to other bovine breeds, other ungulates, or other mammals. The levels of organization of human variation in the analysis of molecular variance of Rosenberg et al. (2002) provide another example. Restricting consideration to level L=2, one might regard the product (6) as the analog of $\left(1-F_{IT}\right)$:


(10) $$\;\begin{array}{rl} 1-G_{IT}^{*} & =(1-G_{1}^{*})(1-G_{2}^{*})=\frac{h_{0}}{h_{2}}\\ & =\frac{1-f}{\left(1-g_{w})(1/d)+(1-g_{b})(1-1/d\right)}+O(1/N). \end{array}$$

Under the identification of $G_{1}^{*}$ with $F_{IS}$ and $G_{2}^{*}$ with $F_{ST}$, this expression recovers the analog of Wright’s iconic partitioning (4):


$$1-F_{IT}=(1-F_{IS})(1-F_{ST}).$$

#### Relative Scaling

In the case study of the Shorthorn cattle (Fig. 1), Wright describes $F_{IT}$ as the correlation between uniting gametes relative to the base population of 1780. Here, each $G_{i}^{*}$ measure (5) is explicitly defined relative to lower levels within the same population. In the extensive literature addressing the *F*-statistics, the nature of scaling or even whether all components are scaled has not always been clear.

Cockerham (1967, 1969, 1973) addressed relationships among the *F*-statistics, the partitioning of correlations, and probabilities of IBD:

I found in the treatment of a single hierarchy of subpopulations … that it was only necessary to define two correlations—simply, that between genes within individuals, *F*, and that between genes of different individuals in the same population, $\bar{\Theta }$. Another correlation, entirely a function of these two, is that between genes within individuals *within* subpopulations

$$f=(F-\bar{\Theta })/(1-\bar{\Theta }).$$

These three correlations manipulate just as the *F*-statistics, and obviously $F=F_{IT}$, $f=F_{IS}$ and $\bar{\Theta }=F_{ST}$ for all intents and purposes. Another distinction is that *F* and $\bar{\Theta }$ have not been defined relative to some standard, although they become so in practice and $F_{IT}$ and $F_{ST}$ are defined relative to a total. (Cockerham 1973, p. 681)

Chesser (1991, p. 439) indicates that Cockerham’s (1969, 1973) identification of $F_{IT}$ with *f* tacitly assumes the absence of IBS among genes at the highest level of the hierarchy. In the hierarchical analysis described here (7),


$$G_{IT}^{*}=1-h_{0}=f,$$

only if genes at level *L* are constrained to be non-IBS ($h_{L}=1$). In general, Equation (6) suggests that at all levels, the analogs of Nei’s *G*-statistics correspond to ratios of non-IBS probabilities, without constraints on their values.

To confirm Wright’s (1977) description of $F_{ST}$ as the correlation between uniting gametes in hypothetical offspring produced by a single generation of random mating, I determine the components of the partitioning described in (10) for those offspring. Wright (1921, p. 119), as well as Malécot (1969) and Jacquard (1974), noted that random union of gametes implies that uniting gametes correspond to gametes randomly sampled from the parental gamete pool:


$$\tilde{\;f}=g_{w}$$

for the tilde indicating values for the hypothetical offspring. Substitution of this expression into (8) produces


$$1-{\tilde{G}}_{1}^{*}=1,$$

and substitution into (10):


$$\begin{array}{rl} 1-{\tilde{G}}_{IT}^{*} & =\frac{1-g_{w}}{\left(1-{\tilde{g}}_{w})(1/d)+(1-{\tilde{g}}_{b})(1-1/d\right)}+O(1/N)\\ & =\frac{1-g_{w}}{\left(1-g_{w})(1/d)+(1-g_{b})(1-1/d\right)}+O(1/N), \end{array}$$

which reflects the assumption that changes of ${\tilde{g}}_{w}$ and ${\tilde{g}}_{b}$ from their counterparts a single generation earlier are O(1/N). These expressions confirm that the overall relationship among the hypothetical offspring corresponds to $G_{ST}^{*}$ before the production of those offspring:


$${\tilde{G}}_{IT}^{*}=G_{ST}^{*}.$$

## IBS Probabilities in Subdivided Populations

This section presents explicit expressions for probabilities of IBS between pairs of genes within individuals (*f*), between individuals within demes ($g_{w}$), and between demes ($g_{b}$) under Wright’s (1943) island model with regular inbreeding and also a two-deme model shown by Weir and Goudet (2017) to yield negative indices of population structure.

### Island Model with Regular Inbreeding

Under Wright’s island model with *d* demes, each with *N* diploid reproductives, a gene in a given deme descends in the immediately preceding generation from a gene in any other deme at backward migration rate *m*. All lineages undergo mutation at rate *u* per generation.

To facilitate the exploration of the IBS-based approach and the hierarchy of population structure it implies (section “Hierarchical Structure”), the model analyzed here also includes regular inbreeding (partial self-fertilization) and the *K*-allele Jukes–Cantor (1969) model of mutation as incorporated into Takahata’s (1983) analysis of Nei’s $G_{ST}$. Under the *K*-allele model, a mutational event in a lineage changes its allelic class to any of K−1 alternative allelic classes with uniform probability. It provides a simple mechanism for generating IBS apart from IBD.

Reproduction by an individual begins with the production of an egg cell, which is fertilized either by another of its own gametes (with probability *s*) or by a gamete sampled from the gamete pool of the deme in which it resides (with the complement probability). Under the assumption that gamete production is not limiting, self-fertilization by an individual has no effect on its contribution to the local gamete pool. Accordingly, a pair of genes randomly sampled from distinct individuals residing in the same deme derive from the same reproductive in the parental generation with probability 1/N, for *N* the number of reproductive individuals, irrespective of the rate of selfing. The probabilities that a random offspring is uniparental (uniting gametes contributed by a single individual) or biparental (uniting gametes contributed by distinct individuals) correspond, respectively, to


(11) $$\begin{array}{cc} & \sigma =s+(1-s)/N,\\ & 1-\sigma =(1-s)(1-1/N). \end{array}$$

Per-generation rates of mutation (*u*) and backward migration (*m*) are assumed to be of order 1/N, with selfing and outcrossing occurring on the much shorter timescale of generations:


(12) s,(1−s)≫u,m,1/N.

**Fig. 2.**
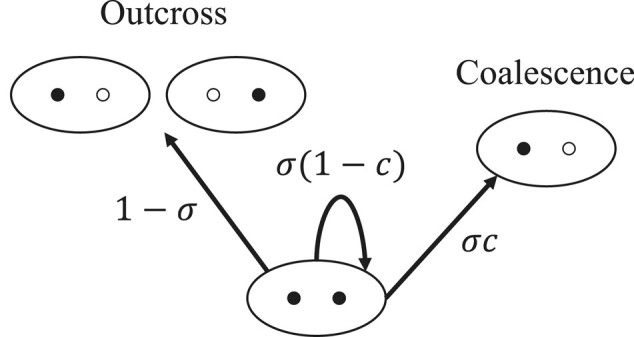
Lineages of complements (filled dots) borne by a single individual. Open dots depict homologs not known to be ancestors of the focal lineages.

Coalescence between a random pair of autosomal genes held by distinct individuals residing in the same deme requires that their ancestral lineages trace back to the same reproductive in the immediately preceding generation. Given parent-sharing, the lineages either derive from the same complement in the shared parent, with probability *c*, or descend from distinct complements, with probability (1−c). Even though


c=1−c=1/2,

these events are distinguished for clarity. Figure 2 depicts the ancestry of complements held by a single individual (filled dots), with open dots representing homologs held by ancestors. With probability (1−σ), the shared parent of the two lineages is biparental, with the lineages descending from distinct individuals in the preceding generation (outcross). Otherwise, with probability *σ*, the shared parent is uniparental, with the lineages once again either coalescing (probability *c*) or remaining distinct (probability 1−c) in the immediately preceding generation. Of the two absorbing states (outcross and coalescence), this process resolves to outcross with probability


(13) $$\frac{1-\sigma }{1-\sigma +\sigma c}$$

and to coalescence with the complement probability. One of the many definitions of effective number ($N_{e}$) addresses the rate of coalescence (Ewens 1982; Crow and Denniston 1988). Using that a pair of genes sampled from distinct individuals residing in the same deme share a parent with probability 1/N, the rate of coalescence corresponds to


(14*a*) $$\begin{array}{rl} \frac{1}{2N_{e}}= & \frac{c}{N}+\frac{1-c}{N}\frac{\sigma c}{1-\sigma +\sigma c}=\frac{1}{2N(1-s/2)}\\ & +O(1/N^{2}). \end{array}$$

This expression is consistent with Pollak’s (1987, p. 354) definition of effective number under partial selfing in populations without subdivision. The relative rate of coalescence corresponds to


(14*b*) $$\Delta =N/N_{e}=1/(1-s/2).$$

This expression indicates that regular inbreeding (s>0) increases the rate of coalescence (Δ>1) by reducing effective number ($N>N_{e}$).

In the model under consideration (12), resolution of the process depicted in Fig. 2 occurs virtually instantaneously relative to mutation and migration. In the absence of those events, lineages that trace back to a shared parent are not IBS only if they resolve to separation in distinct individuals (13) and the ancestral lineages are not IBS:


(15) $$\begin{array}{rl} 1-f & =\;(1-g_{w})\frac{1-\sigma }{1-\sigma /2},\\ \frac{1-f}{1-g_{w}} & =\;\frac{1-s}{1-s/2}+O(1/N). \end{array}$$

While $g_{w}$ here actually represents the IBS probability at least one generation prior to the point in time associated with *f*, we treat the probabilities as contemporary because changes in $g_{w}$ during the short interval required for resolution are of order 1/N. Expression (15) provides the component of the hierarchy identified with $G_{IS}$ (8):


(16) $$\begin{array}{rl} 1-G_{IS}^{*}=\frac{h_{0}}{h_{1}} & =\frac{1-f}{1-g_{w}}+O(1/N)=\frac{1-s}{1-s/2}+O(1/N),\\ & \;G_{IS}^{*}=\frac{s/2}{1-s/2}+O(1/N). \end{array}$$

This classical expression for $F_{IS}$ has been derived on numerous occasions, from models with mutation (Pollak 1987) and without mutation (Haldane 1924; Li 1976, Chapter 13).

While the process portrayed in Fig. 2 resolves over the course of a few generations, IBS probabilities between genes sampled from the same deme $\left(g_{w}\right)$ or from distinct demes $\left(g_{b}\right)$ evolve on the much longer timescale determined by mutation, migration, and coalescence (12). Appendix A describes the derivation of the steady-state values of these quantities. Substitution of those expressions (A.2) into (9) produces


(17) $$\begin{array}{r} G_{ST}^{*}=\frac{\Delta }{\Delta +\frac{d}{d-1}\left[M\frac{d}{d-1}+\theta \frac{K}{K-1}\right]}+O(1/N), \end{array}$$

in which *M* denotes the scaled rate of backward migration and *θ* the scaled rate of mutation (A.1). Here, the relative rate of coalescence *Δ* (14b) captures the entire effect of regular inbreeding: the reduction in effective number within demes from *N* to N(1−s/2). Furthermore, Equation (17) indicates that $G_{ST}^{*}$ declines uniformly with an increases in rates of migration (*M*) and mutation (*θ*). This trend mirrors previous findings that migration and mutation both tend to reduce identity within demes (Spieth 1974; Slatkin 1982; Strobeck 1987).

Under the assumptions of random mating within demes (Δ=1), large deme number (d→∞), the infinite-allele model of mutation (K→∞), and the absence of mutation (θ=0), $G_{ST}^{*}$ (17) reduces to a very well-known expression:


$$\frac{1}{1+M}$$

(Wright 1951). Although the ultimate fate of the metapopulation is fixation on one of the alleles under any positive rate of migration among demes (M>0), Wright’s analyses did not incorporate mutation. In contrast, Takahata (1983) derived the expectation of Nei’s $G_{ST}$ using the stationary distribution of allele frequencies obtained from a diffusion approximation that explicitly incorporates mutation. His results, which are consistent with (17), indicate that the expectation of Nei’s $G_{ST}$ depends on the scaled rate of mutation (*θ*).

### Negative Indices of Population Structure

Weir and Goudet (2017) directly address the role of mutation in a two-deme model, allowing unequal migration rates and deme sizes. Their numerical analysis of the underlying recursions governing evolutionary change identifies parameter combinations that yield negative deme-specific $F_{ST}$. As noted earlier, Wright’s observation that $F_{ST}$ reduces to the (non-negative) Wahlund variance applies to a metapopulation comprising demes among which no migration occurs (Wright 1969, p. 295).

Uyenoyama et al. (2019) obtained analytical expressions for steady-state IBS probabilities in a model similar to Weir and Goudet’s. Appendix B describes the approach and provides the IBS probabilities (B.1) for a pair of genes, both sampled from deme 0 ($g_{w,0}$), both from deme 1 ($g_{w,1}$), and one from each deme ($g_{b}$). Those expressions confirm that all IBS measures evolve to unity in the absence of mutation (θ=0).

For $N_{i}$ is the number of reproductive individuals in deme *i* (i=0,1), *N* denotes the average number of reproductives within demes,


$$N=(N_{0}+N_{1})/2,$$

and *r* denotes the proportion of reproductives that reside in deme 0:


(18) $$r=N_{0}/(N_{0}+N_{1}).$$

For effective number $N_{e}$ defined as the rate of coalescence of a pair of lineages sampled from the same deme (compare (14a)):


$$N_{e}=N,$$

irrespective of the relative sizes of the demes. In fact, this property holds for any number of demes (Appendix B). As in the definition of *θ* and *M* in the island model (A.1), the $M_{i}$ correspond to the limits of the product of backward migration rates ($m_{i}$) and *N*:


(19) $$M_{i}=\underset{\underset{N\to \infty }{m_{i}\to 0}}{\text{lim}}4Nm_{i}.$$

The analysis presented here restricts consideration to rates of mutation and migration of the order of the inverse of the average deme size (12). In contrast, Fig. 2 of Weir and Goudet (2017) appears to indicate that much of the parameter space associated with negative deme-specific $F_{ST}$ includes migration rates of greater order. In addition, note that the demes are labeled 0 and 1 in Uyenoyama et al. (2019) and 1 and 2 in Weir and Goudet (2017).

In terms of the notation used here, Weir and Goudet’s (2017) measure of population structure specific to deme 0 corresponds to


(20*a*) $$\beta_{WT}^{0}=\frac{g_{w,0}-g_{b}}{1-g_{b}},$$

for $g_{w,0}$ is the IBS probability between a pair of genes sampled from deme 0 and $g_{b}$ is the IBS probability between a gene sampled from deme 0 and a gene sampled from deme 1. An analogous deme-specific measure based on $G_{ST}^{*}$ (9) might be defined as


(20*b*) $$\begin{array}{rl} 1-G_{ST,0}^{*} & =\frac{1-g_{w,0}}{r(1-g_{w,0})+(1-r)(1-g_{b})},\\ G_{ST,0}^{*} & =\frac{\left(1-r)(g_{w,0}-g_{b}\right)}{r(1-g_{w,0})+(1-r)(1-g_{b})}. \end{array}$$

These expressions imply


$$\beta_{WT}^{0}-G_{ST,0}^{*}=\frac{\left(g_{w,0}-g_{b})r(1-g_{w,0}\right)}{\left[r(1-g_{w,0})+(1-r)(1-g_{b})](1-g_{b}\right)}.$$

While these measures agree only in the absence of population structure ($g_{w,0}=g_{b}$), both assume negative values only if the IBS probability between genes sampled from different demes exceeds that between genes sampled from deme 0 ($g_{b}>g_{w,0}$). In all cases in which the measures differ, the absolute value of $\beta_{WT}^{0}$ exceeds the absolute value of $G_{ST,0}^{*}$, irrespective of sign.

From (B.1), negativity of the deme-specific indices of population structure (20) requires that *r* (18), the proportion of reproductives that reside in deme 0, exceed


(21) $$r_{\min}=\frac{1+M_{0}+2(M_{1}+\theta )}{2(M_{0}+M_{1}+\theta )}>1/2.$$

This qualitative behavior appears consistent with the trends depicted in Fig. 2 of Weir and Goudet (2017), in which most parameter combinations with negative $F_{ST}$ include larger sizes for the specified deme ($N_{1}>N_{2}$). Negative indices can also arise for values of $N_{1}$ smaller than $N_{2}$, provided that backward migration rates in deme 2 are sufficiently large ($m_{2}>0.15$). For $N_{2}=1,000$, backward migration of this magnitude would suggest scaled migration rates (our $M_{i}$) in excess of 150, values which may be inconsistent with our assumption of rates of migration, mutation, and coalescence of similar orders of magnitude (12).

By analogy to soft selection (Wallace 1975), one might consider *soft migration*, under which migrant number ($M_{i}$) is relatively unconstrained by local deme size. In this case, $r_{\min}$ (21), the minimum relative size of deme 0 that implies non-positive $G_{ST,0}^{*}$, declines uniformly with increasing $M_{0}$. This trend signifies that the conditions for greater between-deme than within-deme similarity ($g_{b}>g_{w,0}$) become less stringent as the number of newly migrated genes increases. This behavior is qualitatively consistent with the negative slope of the red curve in Fig. 2 of Weir and Goudet (2017). That large local deme size and high numbers of migrants reduce within-deme identity ($g_{w}$) relative to between-deme identity ($g_{b}$) is consistent with previous studies of the island model (e.g. Slatkin 1982; Strobeck 1987).

Alternatively, *hard migration* might require equality between the numbers of new migrant genes in the two demes:


(22) $$\begin{array}{rl} N_{0}m_{0} & =\;N_{1}m_{1},\\ rM_{0} & =\;(1-r)M_{1}. \end{array}$$

Imposition of this constraint on (21) indicates that both analogs of deme-specific $F_{ST}$ (20) never take negative values ($r>r_{\min}$ is never satisfied).

## Discussion

This analysis addresses the effect of the nature of the mutation process on Nei’s $G_{ST}$ and related measures (6), providing expressions for the probability that a pair of genes share their allelic class. The shift in perspective from IBD to identity-by-state (IBS) obviates the need to specify an ancestral base population and to account for subsequent changes in allele frequency or other characteristics (Nei 1977). It also demands explicit characterization of the mutation process.

A rephrasing in terms of IBS probabilities of Nei’s (1977) hierarchy of diversity measures offers a resolution to the long-standing confusion surrounding the meaning of $F_{ST}$ as a “relative” measure, as noted by Weir (2012) in the Introduction. The measure of association (5) between a pair of genes sampled at any level of the hierarchy corresponds to the non-IBS probability *relative to* the non-IBS probability at the level immediately below it.

### Locus-Specific Effects on Population Structure

Wright intended that $F_{ST}$ (denoted as *F* in the following quote) primarily reflect genome-wide effects of population structure:

It should be noted that if the coefficient *F* is used for the purpose for which it was originally introduced, the description of population structure, it cannot take cognizance of rates of mutation or selection since these are specific for each locus. (Wright 1952, p. 312)

Even so, the level of diversity observed segregating at a locus in natural populations generally reflects the rate of mutation, a quantity that is often locus-specific. Weir (2012) has made the important point that Wright’s $F_{ST}$ is not a statistic but rather a parameter of the population. From this perspective, ensuring that patterns of variation at multiple loci contribute to estimates of the same quantity may require accounting for the mutation process.

Some authors have found the dependence of $F_{ST}$ on the spectrum of allele frequencies in a sample of genes problematic (e.g. Jost 2008). One way to address this concern entails adopting an index of population structure that is less sensitive to allele frequencies. Slatkin’s $F_{ST}$ (1991, distinguished here by an asterisk) is defined not in terms of allele frequencies, but rather the expected ages of the most recent common ancestor (MRCA) of randomly sampled pairs of genes:


(23) $$F_{ST}^{*}=\frac{E(T)-E(T_{s})}{E(T)},$$

for $T_{s}$ is a random variable representing the age of the MRCA of a pair sampled from the same deme and *T* is the age of the MRCA of a pair sampled from the population at large without regard to population structure. Inferences based on empirical data still of course require the observation of genetic variation, but estimates can be obtained in cases in which genetic distance between genes increases linearly with time since their separation. Slatkin (1991) noted that $F_{ST}^{*}$ converges to Nei’s $G_{ST}$ as the rate of mutation vanishes (u→0).

Another approach entails directly confronting the origin and nature of the genetic variation that serves as the basis for inferences about population structure. In his analysis of Nei’s $G_{ST}$, Takahata (1983) derived expectations of diversity at the stationary distribution of allele frequencies implied by the mechanism of mutation and population structure. While accounting for mutation is less common in the context of pedigree analysis, its importance as the ultimate source of genetic variation has of course long been acknowledged. Malécot’s (1969) expression for the IBD probability in unstructured populations,


$$\underset{\underset{u\to 0}{N\to \infty }}{lim}\frac{1/2N}{2u+1/2N}=\frac{1}{\theta +1},$$

agrees with the expression from the Ewens Sampling Formula (ESF; Ewens 1972) for samples of size 2. Significantly, Thompson (2008) invoked the ESF, which depends on the scaled mutation rate, to characterize the base population from which IBD probabilities among four genes are determined. Pollak (1987) explicitly included mutation in his analysis of the *F*-statistics under partial selfing.

Within the context of the hierarchy of IBS probabilities considered here (6), mutation as well as migration affects population structure by reducing identity within demes. Expressions for pairwise IBS probabilities at steady state under the island model (17) indicate lower levels of population structure in regions of the genome characterized by higher mutation rates (*θ*). This pattern is consistent with trends observed in empirical studies suggesting that high-diversity loci, including microsatellites, tend to yield lower values of $F_{ST}$ than low-diversity loci, including biallelic single nucleotide polymorphisms (Jakobsson et al. 2013, and references therein). Furthermore, the nature of the process of mutation as well as its rate influences $G_{ST}^{*}$. Under the simple *K*-allele mutation model, the effect of mutation increases as the number of possible allelic classes declines. For K=4, the magnitude of the term involving *θ* in $G_{ST}^{*}$ (17) increases by a factor of 4/3 over the infinite-alleles model.

### *F*_*ST*_ as an Index of Distance

Among the broad array of empirical uses of $F_{ST}$, a prominent one is an index of genetic distance between populations. For example, it forms the basis of a striking trend supporting the serial founder hypothesis for the origins of humanity (Ramachandran et al. 2005).

Also defined in terms of genetic diversity, Nei’s (1972) distance is closely related to $F_{ST}$. In the context of the two-deme model discussed here and in Weir and Goudet (2017), the analog of this measure in terms of IBS probabilities corresponds to


(24) $$D^{*}=-\frac{1}{2}\left[\log\;\left(\frac{g_{b}}{g_{w,0}}\right)+\log\;\left(\frac{g_{b}}{g_{w,1}}\right)\right],$$

in which $g_{b}$ denotes the probability of IBS between a gene sampled from deme 0 and a gene sampled from deme 1 and $g_{w,i}$ is the IBS probability between a pair of genes sampled from distinct individuals in deme *i* (i=0,1). Other authors (see Slatkin 1982 and references therein) have also proposed expressions of the form $g_{b}/g_{w,i}$ as measures of correlation among demes induced by migration.

Although one might expect a measure of distance to satisfy the triangle inequality, Nei (1972) noted that his *D* does not necessarily have this property. Arbisser and Rosenberg (2020) showed that in the case of a biallelic SNP that occurs in distinct frequencies across the three subpopulations, $F_{ST}$ always fails to satisfy the triangle inequality.

Population- and locus-specific measures of $F_{ST}$ may provide a means of identifying populations or genomic regions with unusual selective or demographic histories (Weir et al. 2005). Weir and Goudet (2017) have noted a growing literature on model-specific Bayesian methods designed to detect outlier values of $F_{ST}$, perhaps indicative of selection at particular loci. In this case, accounting for the mutation process becomes all the more important as the relative effects of migration and mutation may well vary across loci.

In a two-deme model with mutation explicitly represented, Weir and Goudet (2017) determined conditions under which deme-specific measures (20) can assume negative values, even in the absence of selection. Negativity reflects higher probability of IBS in between-deme comparisons than within-deme comparisons ($g_{b}>g_{w,0}$). Examination of the analytical expressions for the IBS probabilities obtained by Uyenoyama et al. (2019) for a similar two-deme model ((21), Appendix B) indicates that factors that tend to reduce $g_{w,0}$ include high local deme sizes (large $N_{0}$) and high numbers of newly arrived migrants (large $M_{0}$). In addition, the nature of migration affects whether negative $\beta_{WT}^{0}$ and $G_{ST,0}^{*}$ can arise. Under conservative or hard migration, which requires equality between the numbers of new migrant genes in the two demes (22), the indices of population structure are always positive at steady state. Unlike deme-specific measures of $F_{ST}$ (20), the analog of Nei’s distance (24) is always positive in this two-deme model.

## Data Availability

No new data were generated or analyzed in support of this research.

## Appendix A. Wright’s Island Model with Inbreeding

Wright’s (1943) island model describes a population comprising *d* demes with identical characteristics. Hudson (1990) derived IBS probabilities for a pair of genes sampled from a single deme and from distinct demes. Presented here are modifications to incorporate partial selfing (Fig. 2) and the *K*-allele model of mutation.

As noted in section “Island Model with Regular Inbreeding”, the process depicted in Fig. 2 resolves on the timescale of generations, virtually instantaneously relative to the processes of mutation, migration, and coalescence. Accordingly, Equation (15) gives the relationship between $h_{0}=(1-f)$ and $h_{1}=(1-g_{w})$, and one need to determine only the non-IBS probabilities between genes sampled from a single deme $\left(1-g_{w}\right)$ or from distinct demes $\left(1-g_{b}\right)$ to extend Hudson’s analysis.

In the genealogical history of a pair of genes, mutation occurs in at least 1 lineage at rate

$$1-(1-u{)}^{2},$$

at least 1 lineage undergoes backward migration at rate

$$1-(1-m{)}^{2},$$

and coalescence occurs between a pair of lineages residing in the same deme at rate

Δ/2N,

where *Δ* is the relative rate of coalescence (14b). Assuming independence among the events and exponentially distributed waiting times, the probability that the most recent event is mutation, for example, corresponds to

$$\frac{1-(1-u{)}^{2}}{1-(1-u{)}^{2}+1-(1-m{)}^{2}+\Delta /2N}.$$

Ignoring terms of $O(u^{2})$ or smaller and taking the limits

(A.1)
$$\begin{array}{cc} \theta & \;=\underset{\underset{u\to 0}{N\to \infty }}{\text{lim}}4Nu,\\ M & \;=\underset{\underset{m\to 0}{N\to \infty }}{\text{lim}}4Nm, \end{array}$$

reduces this expression to

$$\text{lim}\frac{2u}{2u+2m+\Delta /2N}=\frac{\theta }{\theta +M+\Delta }.$$

Similarly, the probability that the most recent event corresponds to migration is

$$\frac{M}{\theta +M+\Delta },$$

and to coalescence,

$$\frac{\Delta }{\theta +M+\Delta }.$$

The probability that a pair of genes sampled from distinct individuals residing in the same deme are IBS corresponds to

$$g_{w}=\frac{\theta (1-g_{w}^{\prime })/(K-1)+Mg_{b}^{\prime }+\Delta }{\theta +M+\Delta },$$

in which the prime indicates the state of the lineages just prior to the most recent evolutionary event. Coalescence between the pair terminates the genealogy in the MRCA. For backward migration as the most recent event, the ancestral state corresponds to an IBS pair of genes in distinct demes, which occurs with probability $g_{b}^{\prime }$. For mutation as the most recent event, the ancestral lineages must reside in the local deme. Those lineages are IBS with probability $g_{w}^{\prime }$ and non-IBS with probability $\left(1-g_{w}^{\prime }\right)$. While a single mutation in an IBS pair cannot generate the descendant state, a non-IBS pair can become IBS through a mutation that transfers one gene into the allelic class of the other, which occurs with probability 1/(K−1), where *K* is the number of allelic classes.

For a gene pair sampled from distinct demes, the most recent evolutionary event cannot correspond to coalescence, which can occur only between lineages residing in the same deme. The probability that a pair of genes sampled from distinct demes are IBS corresponds to

$$g_{b}=\frac{\theta (1-g_{b}^{\prime })/(K-1)+M\{g_{w}^{\prime }/(d-1)+g_{b}^{\prime }[1-1/(d-1)]\}}{\theta +M}.$$

A backward migration event that transfers one gene of the descendant pair into the deme of the other gene implies an ancestor comprising IBS genes in the same deme, which occurs with probability $g_{w}^{\prime }$. Any other origin of the migrating lineage preserves the residence of the lineages in distinct demes.

Solution of these expressions at steady state ($g_{w}^{\prime }=g_{w},g_{b}^{\prime }=g_{b}$) produces

(A.2*a*)
$$1-g_{w}=\frac{\theta \left[\theta \frac{K}{K-1}+M\frac{d}{d-1}\right]}{D},$$

(A.2*b*)
$$1-g_{b}=\frac{\theta \left[\Delta +\theta \frac{K}{K-1}+M\frac{d}{d-1}\right]}{D},$$

in which

D=ΔM/(d−1)+[Δ+Md/(d−1)+θK/(K−1)]θK/(K−1).

It is simple to show that this equilibrium state is locally attracting.

That both non-IBS probabilities (A.2) are proportional to the mutation parameter *θ* reflects that diversity at steady state requires mutation. In subdivided populations (d>1) reproducing through random union of gametes (s=1/N), the expressions obtained from (A.2) for IBS probabilities $g_{w}$ and $g_{b}$, respectively, reduce to Takahata’s (1983) expected gene identities within ($J_{0}$) and between ($J_{1}$) demes, provided that the rightmost vector in his Equation (7) is replaced by

$$\left(\begin{array}{c} 1+4\theta^{*}\\ 4\theta^{*} \end{array}\right).$$

The absence of the four in the lower element appears to be a typographical error in that publication. A labeled coalescent argument (Karlin and McGregor 1972; Uyenoyama et al. 2019) also yields the non-IBS expressions (A.2).

## Appendix B. Two-Deme Model

Uyenoyama et al. (2019) addressed the analog of the ESF (Ewens 1972) in a two-deme population with random mating within demes. Their recursion determines the allele frequency spectrum under the infinite-alleles model of mutation inductively, for progressively larger sample sizes. For samples of size 2, their Equation (15) provides the IBS probabilities for a pair of genes sampled within a given deme or a pair sampled from different demes.

In this two-deme model, deme *i* (i=0,1) comprises $N_{i}$ diploid reproductives, with

$$r=N_{0}/(N_{0}+N_{1})$$

(18) the proportion of reproductives residing in deme 0. Deme *i* has backward migration rate $m_{i}$. In both demes, mutation occurs at rate *u* at the autosomal locus from which the sample derives. Migration and mutation occur at rates of order 1/N, where *N* is the average number of reproductives:

$$N=(N_{0}+N_{1})/2.$$

For a *d*-deme model, with

$$r_{i}=\frac{N_{i}}{\sum_{j}N_{j}},$$

we retain the definition of *N* as the unweighted arithmetic mean of the number of reproductives across demes. Effective number $N_{e}$, defined as the rate of coalescence of a pair of lineages sampled from the same deme, corresponds to

$$\begin{array}{rl} \frac{1}{2N_{e}} & =\sum_{i}\frac{r_{i}}{2N_{i}}\\ & =\frac{d}{2\sum_{j}N_{j}}. \end{array}$$

This expression implies that irrespective of the number of demes or their relative sizes, the effective number again corresponds to *N*, the unweighted average number of reproductives across demes:

$$N_{e}=\frac{\sum_{j}N_{j}}{d}=N.$$

Accordingly, the relative coalescence rate (14b) corresponds to

Δ=1,

reflecting random mating within demes.

For the two-deme case (d=2), Equation (15) of Uyenoyama et al. (2019) provides the IBS probabilities for a pair of genes sampled from deme 0 ($g_{w,0}$), from deme 1 ($g_{w,1}$), and from both demes ($g_{b}$):

(B.1)
$$\begin{array}{cc} & g_{w,0}=M_{0}+M_{1}+2[r(M_{0}^{2}+\theta )+(1-r)(M_{1}^{2}+\theta )\\ & \;\;\;+\;(1-r)\theta (M_{0}+3M_{1}+2\theta )]/H,\\ & g_{b}=M_{0}+M_{1}+2[rM_{0}(M_{0}+\theta )+(1-r)M_{1}(M_{1}+\theta )]/H,\\ & g_{w,1}=M_{0}+M_{1}+2[r(M_{0}^{2}+\theta )+(1-r)(M_{1}^{2}+\theta )\\ & \;\;\;+\;r\theta (3M_{0}+M_{1}+2\theta )]/H, \end{array}$$

for

$$\begin{array}{rl} H & =(1+2\theta )(M_{0}+M_{1}+2\theta )+2\{rM_{0}(M_{0}+2\theta )\\ & \;+(1-r)M_{1}(M_{1}+2\theta )\\ & \;+2r(1-r)\theta (M_{0}+M_{1}+\theta )(M_{0}+M_{1}+2\theta )\}, \end{array}$$

with *θ* given in (A.1) and the $M_{i}$ in (19). As expected, the entire population becomes monomorphic ($g_{w,0}=g_{b}=g_{w,1}=1$) in the absence of mutation (θ=0).
